# Titers, Prevalence, and Duration of SARS-CoV-2 Antibodies in a Local COVID-19 Outbreak and Following Vaccination

**DOI:** 10.3390/vaccines9060587

**Published:** 2021-06-02

**Authors:** Jodi F. Hedges, Macy A. Thompson, Deann T. Snyder, Amanda Robison, Matthew P. Taylor, Mark A. Jutila

**Affiliations:** Department of Microbiology and Cell Biology, Montanta State University, Bozeman, MT 59717, USA; 14macythompson@gmail.com (M.A.T.); deann.snyder@montana.edu (D.T.S.); amanda.robison@montana.edu (A.R.); mptaylor@montana.edu (M.P.T.); mark.jutila@montana.edu (M.A.J.)

**Keywords:** anti-RBD IgG, antibodies, SARS-CoV-2, COVID-19, neutralization, duration, asymptomatic, vaccine

## Abstract

Information concerning the development of neutralizing antibodies and their duration will be critical to establishing herd immunity for COVID-19. We sought to evaluate SARS-CoV-2 spike protein receptor-binding domain (RBD)-specific antibodies, their duration, and capacity for SARS-CoV-2 neutralization in volunteers while the pandemic spread within our community starting in March 2020. Those participants with the highest starting titers had the longest-lasting response, up to 12 months post-diagnosis. SARS-CoV-2 neutralization capacity was correlated with anti-RBD antibody levels. The majority of our participants with confirmed COVID-19 diagnosis had very mild or asymptomatic infections. We also detected low and largely non-neutralizing anti-RBD IgG titers in a few participants with no known COVID-19 diagnosis. Finally, we found that antibody responses induced by vaccination were significantly higher than those induced by natural infection. Thus, our study suggests that vaccination is still critical even for those naturally infected or diagnosed with COVID-19.

## 1. Introduction

The novel coronavirus severe acute respiratory syndrome coronavirus 2 (SARS-CoV-2) is responsible for the current global pandemic, COVID-19. Despite the concerted research effort, much remains to be understood on both the host and pathogen levels. As vaccines become available, it is critical that we bolster our knowledge of the humoral adaptive immune responses against SARS-CoV-2. Protective immunity induced by infection and vaccination has been shown in multiple instances including in rhesus macaques, mice, and in the COVID-19 outbreak that occurred on a fishing vessel [1,2,3,4,5,6]. More recently data from trials in humans have shown that new mRNA vaccines strongly stimulate antibody responses and protect from infection [7,8]. Additionally, antibody treatments are now approved for use in COVID-19 [9]. In order to achieve effective herd immunity, we must thoroughly understand antibody responses to natural SARS-CoV-2 infection and responses to new vaccines.

A number of reports have presented data on the duration of detectible circulating antibodies for COVID-19. Many studies have assessed patients of varying disease severity and have found that antibody titers and capacity for neutralization are closely associated with disease severity [10,11,12,13,14]. In terms of the duration of the antibody response, most studies followed patients for a number of weeks to months [10,11,12,13,15]. Fewer studies followed the same participants for longer periods, and some have extended findings to the development of memory B cell populations up to eight months after infection [16,17]. Study designs range from a few participants from limited outbreaks [18] to larger numbers of severely ill patients for shorter intervals post-infection [12,19]. Others have done broad population studies on antibody prevalence with minimal in depth or repeat analyses [20]. Few of these studies have involved repeated assessments of antibody levels and SARS-CoV-2 neutralizing capacity for many months in participants with largely mild or no symptoms.

Tests used to measure antibody levels differ between studies, some are certainly less sensitive than others or only reflect the presence or absence of antibodies [21], and these differences likely confound the findings. Few studies broadly tested volunteers with highly sensitive tests and repeatedly sampled the same people. Furthermore, most assays utilize surrogate or pseudovirus neutralization assays that approximate the neutralization capacity of serum for SARS-CoV-2 [12,13,17]. The intent of this study was to determine the extent of antibody detection in the general population and to assess their duration and capacity to neutralize SARS-CoV-2. We employed a highly specific and sensitive assay to measure SARS-CoV-2 spike protein receptor-binding domain (RBD)-specific IgG paired with SARS-CoV-2 neutralization assays. We assessed donors from the local community independent of COVID-19 diagnosis and collected sera monthly for up to 12 months. We also assessed sera from individuals after vaccination (Pfizer/BioNTech or Moderna) for comparison to responses induced by natural infection. Our data suggest that vaccines will be beneficial and necessary even for people who were diagnosed with COVID-19 or tested positive for antibodies, as diagnosis or detection of antibodies does not guarantee robust immunity.

## 2. Materials and Methods

### 2.1. Human Subjects

This study was approved by the Institutional Review Board at Montana State University and in accordance with the Helsinki Declaration. Participants for the study were recruited by way of informative flyers posted in two buildings at Montana State University, and by word of mouth. These included individuals with previous clinical positive or negative SARS-CoV-2 tests, those with suspected COVID-19 symptoms, and those with no diagnosis, history of contact, or symptoms. Informed consent was obtained from all participants who also answered a survey regarding their, age, history of illness, travel, and COVID-19 testing. The mean age of our study population is 41.8 years (95% CI 41 to 42.6) based on 141 participants reporting their ages, which is higher than the average age of 29 years in our county. Twenty-eight serum samples collected prior to 2016, well before the start of the COVID-19 pandemic, were used as negative controls. Participants found to have high anti-RBD IgG antibody titers were asked to return for repeat donations on a monthly basis.

One tube of blood was collected in a BD Vacutainer SST Venous Blood Collection Tube from each patient per sampling day. Tubes were centrifuged at 1150 g for 10 min to separate the serum. One aliquot of serum was delivered directly to our BSL-3 facility for use in virus neutralization assays. The remainder of the serum was treated with 1% Triton-X 100 to inactivate any SARS-Cov-2 virus and aliquoted into smaller sample sizes, which were either frozen at −80 °C or stored at 4 °C for immediate use.

### 2.2. Enzyme-Linked Immunosorbent Assay (ELISA)

An ELISA was performed on the serum to identify RBD-specific IgG antibodies, essentially as previously described [22]. The ELISA plate (Nunc MaxiSorp™, Thermo Fisher, Waltham, MA, USA) was coated with 2 μg/mL of the recombinant SARS-CoV-2 Receptor-Binding Domain (RBD) either obtained from BEI Resources or as a kind gift (from Jesse D.Bloom, Roland Strong and Chance M. Brock, Fred Hutchinson Cancer Research Center) in phosphate-buffered saline (PBS) and incubated at 4 °C overnight. The plate was washed five times with 0.1% Tween-20 in PBS (TPBS). The plate was then blocked with 3% nonfat milk in TPBS for 1 h at room temperature (RT). Serial dilutions were performed with the human serum samples from 1:32 through 1:4096 in 1% nonfat milk in TPBS, while mouse sera were diluted at 1:16. Each ELISA plate included a negative control serum collected before 2016 and a positive control (the same serum from a participant with a confirmed positive COVID-19 diagnosis). Diluted sera were then transferred to the ELISA plate and incubated at RT for 2 h. The plate was then washed five times with TPBS, and secondary goat anti-human IgG (Cat# A18811, Invitrogen) or goat anti-mouse IgG (Southern Biotech Cat.# 1030-05) conjugated to horseradish peroxidase (HRP) was added and incubated for 1 h at RT. The plate was then washed again with TPBS five times, and Sigma-Aldrich o-phenylenediamine dihydrochloride (OPD), or TMB, was added for exactly 10 or 3 min (respectively) before being stopped by 3 molar hydrochloric acid (HCL) or 2N sulfuric acid (H2SO4). The plate was read in an ELISA reader at 490 nm or 450 nm respectively.

### 2.3. SARS-CoV-2 Neutralization Assays

The neutralization titers of human or mouse sera were determined using SARS-CoV-2 under BSL-3 containment essentially as previously described [23]. SARS-CoV-2 (WA/01 strain, BEI Resources) was propagated on Vero E6 cells as previously described [24] and quantified by endpoint titration and plaque assay. The sera were serially diluted from 1:32 to 1:4096 and mixed with 1000 TCID50 of SARS-CoV-2. The mixture was incubated for 1 h then applied to confluent monolayers of low passage Vero E6 cells in a 96 well flat-bottomed plate. The plates were incubated for 48 h then the supernatant fluids were removed, and the cell monolayers were stained with (0.5%) methylene blue in 70% ethanol. The extent of neutralization was determined by observation of monolayer clearance in the well, and neutralization titers are reported as the inverse of the serum dilution that provided 50% protection of the monolayer. Each serum was assessed in duplicate and in at least two separate neutralization assays to confirm the findings. Neutralization titers were expressed as an average of the titers determined for that sample.

### 2.4. Mouse Methods

All animal studies were reviewed and approved by the Montana State University IACUC committee. Male mice were purchased from Jackson Laboratory [B6.Cg-Tg(K18-ACE2)2Prlmn/J, Stock No: 034860]. K18-hACE2 transgenic mice express human ACE2 (hACE2), the receptor used by SARS coronaviruses to gain cellular entry. The K18 promoter directs expression to epithelia, including airway epithelia where infections typically begin. We compared carriers for hACE2 to non-carrier littermates (wild type). Seventeen-week-old mice were lightly anesthetized with isoflurane and oxygen, and given either 10^4^ or 10^5^ PFU SARS-CoV-2 in 30 μL by the intranasal route. Mice were weighed and their health was monitored daily. Mice were euthanized by CO_2_ inhalation 5 or 6 days post-infection. Terminal blood was collected and plasma was separated and aliquoted for use in the neutralization assay or heat-inactivated at 65 °C for 20 min to perform the RBD IgG ELISA.

### 2.5. Statistical Analyses

Statistical analyses were performed using Prism (GraphPad Software, San Diego, CA, USA). To determine the cutoff line, the optical densities (OD) of the negative control samples were averaged and three standard deviations above the negative average were established as the necessary cutoff value a sample must exceed to be considered positive for SARS-CoV-2 RBD IgG, as described previously [22]. ODs were graphed using GraphPad Prism and the area under the curve (AUC) was determined for each sample. The Kolmogorov-Smirnov test (with Dallal-Wilkinson-Liliefor *p*-value) was used to determine if the data formed a Gaussian distribution. Much of the AUC and neutralization data were not normally distributed, thus, they were analyzed with the Mann-Whitney test when comparing only two groups and with the Kruskal-Wallis test with Dunns post-test when comparing multiple groups. Parametric (Pearsons) or nonparametric correlation (Spearman) was used to determine correlation values. Paired data were compared using the Wilcoxon matched-pairs signed-rank test.

## 3. Results

**Figure 1:**
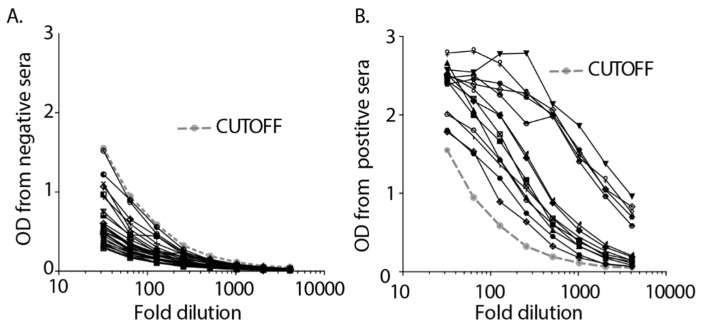
A sensitive and specific ELISA was used to measure antibody levels. (**A**). 28 serum samples collected prior to 2016 were used to determine the cutoff value for antibody levels. (**B**). The first 14 serum samples from participants with confirmed COVID-19 diagnosis served as positive controls for the ELISA and were determined to fall above the cutoff line.

The extent of asymptomatic infection with SARS-CoV-2 in our community (Bozeman, Gallatin County, MT, USA) was of initial concern when COVID-19 started expanding in Montana in March 2020. We began serological testing for the presence of SARS-CoV-2-specific antibodies using an optimized ELISA protocol adapted from Krammer et al. [22,25]. The resulting ELISA was highly specific, with no reactivity to 28 human serum samples collected prior to 2016 (Figure 1A). In contrast, the positive controls, the initial sera from 14 people identified with COVID-19 diagnosis, all had ODs above the calculated cutoff (Figure 1B). These data establish that the ELISA assay used in this study was highly sensitive and specific.

Between April and November of 2020, 168 participants (107 females, 61 males) were enrolled in our study to understand antibody responses to SARS-CoV-2. Thirty-four subjects (18.9%) were diagnosed with COVID-19, or had known recent close contacts but were not tested due to early limited testing availability. Those diagnosed with COVID-19 were separated into two groups, those infected in March 2020 (14 people) and those infected in the second wave of infections starting in July 2020 (20 people). Data concerning the local COVID-19 outbreak is publicly available: https://www.healthygallatin.org/coronavirus-covid-19/, accessed on 31 May 2021. Ten out of the remaining 134 participants were not diagnosed with COVID-19 and had no known contacts, yet had detectable antibody titers. Participants indicated their disease severity on a scale of 0–3 from asymptomatic to severe. The breakdown of antibody detection and disease severity of these participants is presented in Table 1. We determined that most volunteers with unknown COVID-19 diagnosis or interactions (124/134 or 92.5%) were negative for COVID-19 antibodies. Twenty-seven people included in our study self-reported an illness in the 6-month period prior to antibody testing; of these only 4 had IgG AUC levels above the cutoff. Thus, despite many being anecdotally “sure” that they had contracted the virus in January or February before COVID-19 was detected and testing was available regionally, most were anti-RBD IgG negative. Clearly, in addition to COVID-19, there were severe cold strains and flu circulating in our community early in 2020. Our small surveillance study determined that a person with no known exposure or diagnosis was very unlikely to have COVID-19 antibodies.

The majority of participants with a confirmed COVID-19 diagnosis had detectible IgG titers (85.3%, 29/34 people). Thus, a small fraction (14.7%, 5/34) of confirmed positive COVID-19 diagnosed participants did not have detectible IgG levels. The mismatch between COVID-19 diagnosis and antibody titers occurred for the first time in August 2020 and then occurred four more times during the increase in COVID-19 cases in our county in the fall of 2020. Furthermore, the AUCs for these people were far below the cutoff. These data suggest either an increased detection of asymptomatic confirmed positive participants that failed to elicit detectible antibody responses or the possibility of increasing misdiagnosis of SARS-CoV-2 infection that occurred during the increase in testing in the fall of 2020.

**Figure 2:**
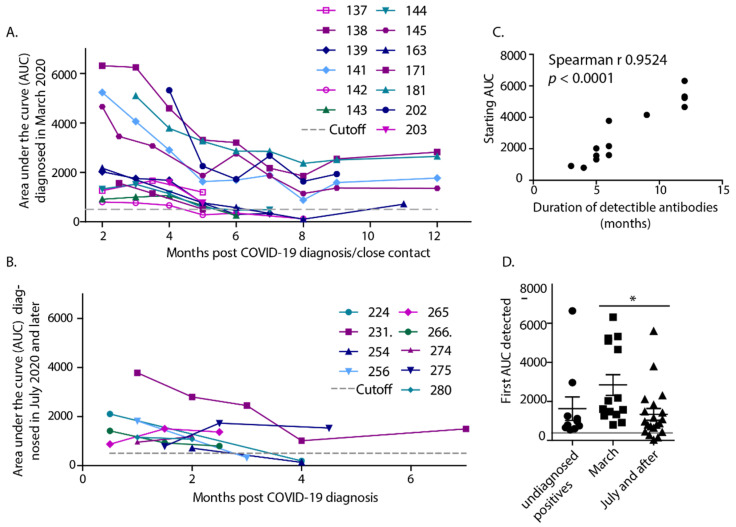
Antibody titers were measured up to 12 months post-infection. (**A**) AUCs were determined monthly post-infection over a longer term for people diagnosed in March and (**B**) over 5 months for participants diagnosed in July or later. (**C**) There was a strong correlation between the starting antibody level (AUC) and the duration of antibodies, from a total of 13 XY pairs, with a Spearman r of 0.9524, *p*-value < 0.0001. (**D**) Initial AUCs of 3 groups, those diagnosed in March, July or after, and those without a diagnosis, but detectible antibody titers. There was a statistical difference between antibody levels from people diagnosed in March compared to those diagnosed in July or after. * *p* < 0.05 as determined by Kruskal-Wallis test with Dunns post-test.

Volunteers with positive ELISA titers were invited to donate serum samples monthly for up to 12 months post-infection, regardless of their COVID-19 diagnosis. RBD-specific antibodies were measured more than once in most participants and were maintained in the majority of participants for 5 months when detection began to wane, with only about 50% of subjects maintaining antibody titers for more than 6 months (Figure 2A,B). Of the 15 people that had antibody assessments monthly for greater than 6 months, only 6 had antibody titers that lasted longer than 6 months. Clearly, those participants with sera collections starting in July lost antibody titers sooner and thus were assessed for a much shorter duration. Alternatively, a few of the individuals with detectible titers did not return for sampling. There were no notable differences in self-reported disease severities between those infected in March, compared to those infected in July or later. Too few ill participants were enrolled to generate a correlation between disease severity and antibody levels. We measured the highest starting titers in participants that were infected in March and found a strong correlation between antibody levels detected initially and the duration of the measurable antibody response (Figure 2C). Infections that occurred in July or after resulted in significantly lower starting antibody levels (Figure 2D). Expanded testing and contact tracing at this later time may have increased the diagnosis of more very mild or asymptomatic infections compared to March. The IgG AUC of participants with no known infection or interaction also appeared lower but was not significantly different from the other groups.

**Figure 3:**
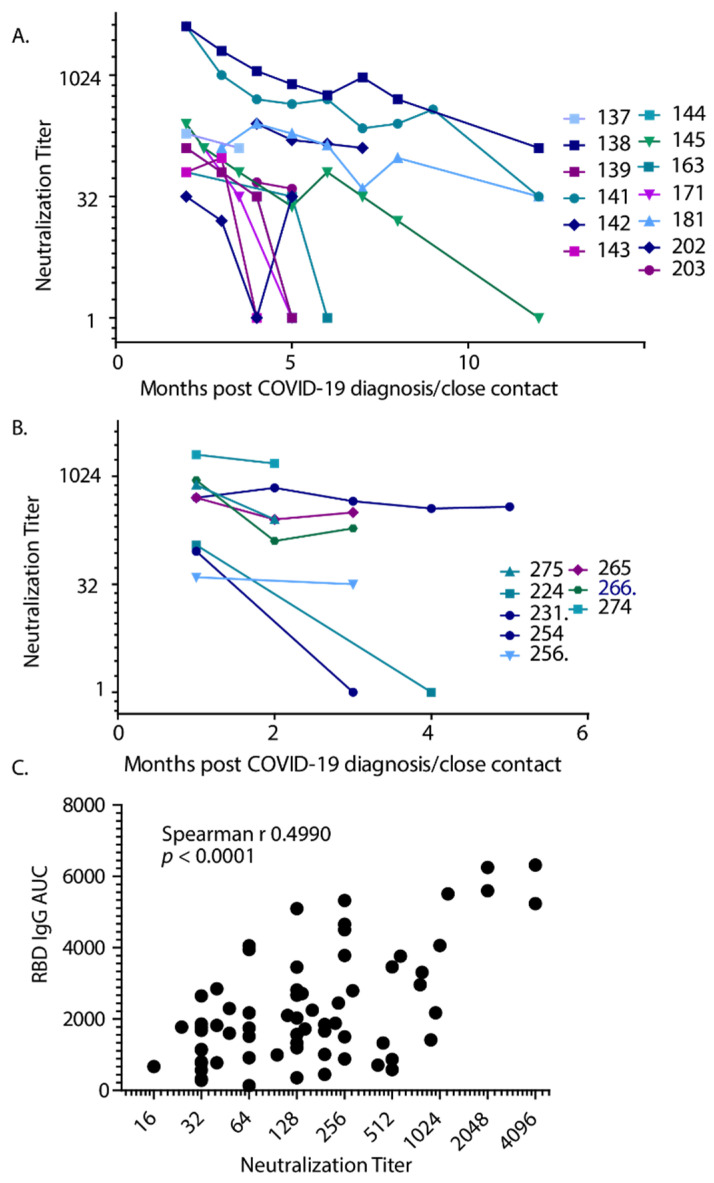
The capacity to neutralize SARS-CoV-2 in vitro was closely related to the RBD IgG AUC. (**A**) Neutralization was determined monthly for participants infected in March 2020 or (**B**) in July or later. (**C**) There was a moderate but reliable correlation between AUC and neutralization titers from 65 XY pairs, with a Spearman r of 0.4990, *p*-value < 0.0001.

We measured the capacity for collected sera to neutralize SARS-CoV-2 for both those infected in March (Figure 3A) and those infected in July and after (Figure 3B). These assays were performed in BSL-3 containment with serum samples that were not inactivated with 1% Triton-X100 and the neutralization titers are reported as the inverse of the serum dilution that provided 50% protection of the monolayer. There was a correlation between anti-RBD IgG AUC and the capacity to neutralize SARS-CoV-2 (Figure 3C). Thus, the results suggest that anti-RBD IgG AUC is a valid predictor of the capacity of serum to neutralize SARS-CoV-2 in vitro, confirming the findings in earlier studies [15,26]. However, serum from one subject was below the cutoff for anti-RBD-IgG but retained the capacity to neutralize the virus (Table 1). Further investigation of this phenomenon is warranted.

**Figure 4:**
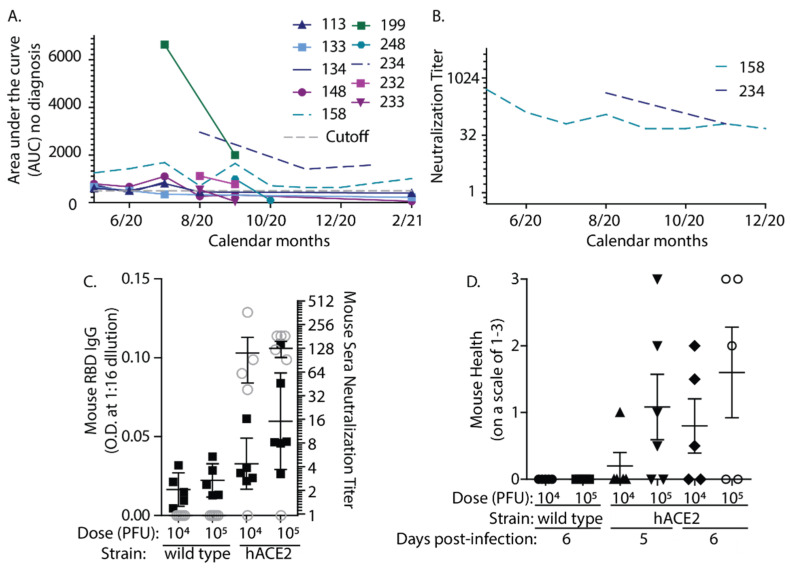
Asymptomatic COVID-19 results in very low titer, largely non-neutralizing antibodies in human subjects with similarities in the mouse model. (**A**). RBD IgG AUC above the cutoff was discovered in 10 participants with no known interaction or diagnosis with COVID-19. (**B**) Only 2 (dashed lines in (**A**) and (**B**)) of these 10 participants had measurable neutralization titers. (**C**). Mice were infected with either 10^4^ or 10^5^ PFU SARS-CoV-2. Mice expressing hACE2 that received the higher dose have slightly higher antibody levels and neutralization titers than those that received a 10-fold lower dose. ELISA titers are represented by black squares, and neutralization titers are represented by gray open circles. (**D**). Mice infected with the higher dose also had greater indications of disease symptoms/morbidity compared to those infected with the lower dose.

We originally hypothesized that testing somewhat random volunteers would help to reveal the extent of asymptomatic infection. While most participants without known COVID-19 diagnosis or close contact were antibody negative, we detected RBD-specific antibodies in 10 participants that had no known interactions or close contacts with confirmed COVID-19 patients (Figure 4A). Of these 10, all but two had no or very mild symptoms. The two participants that reported severe symptoms had either a negative COVID-19 test or the symptoms that occurred well before COVID-19 had been locally detected. These ten participants were assessed over time, and all but two had antibody AUCs that were considered positive upon repeated measures. Although antibodies were measurable by ELISA, only two of these 10 had long-lasting SARS-CoV-2 neutralizing antibodies. In one case, these titers were maintained for more than 6 months (Figure 4B). While there are multiple studies concerning antibodies following hospitalization or confirmed COVID-19 diagnosis, not many studies have assessed antibody levels in people who are undiagnosed with COVID-19. This small subset of ELISA-positive participants without known diagnosis underscores the fact that antibody titers in the absence of known COVID-19 contacts or diagnosis are likely very low and non-neutralizing.

The generation of variable antibody levels depending on infectious dose can be modeled in mice infected with SARS-CoV-2. Lacking the viral receptor, wild type mice do not become infected with SARS-CoV-2, whereas transgenic mice expressing hACE2 demonstrate measurable symptomatic responses [27]. We compared antibody responses after infection with 2 doses of SARS-CoV-2 in transgenic hACE-2 mice. Mice were infected with 10^4^ or 10^5^ PFU of SARS-CoV-2 by the intranasal route. Most mice expressing hACE2 developed measurable neutralizing antibodies after only 6 days of infection, whereas the wild type mice did not (Figure 4C). The hACE2 mice infected with the higher dose generally developed slightly higher antibody titers and more severe symptoms of disease (morbidity) than those receiving the lower dose (Figure 4D). However, some hACE2 mice did not respond to infection and this variation between animals precluded significance. There are many caveats to the interpretation of these experiments and any conclusions on how reflective they might be of responses in humans should be tempered. However, the data suggest that similar to the situation in humans, mice vary in responses to infection, and those that develop less severe disease symptoms can have less robust antibody responses.

**Figure 5:**
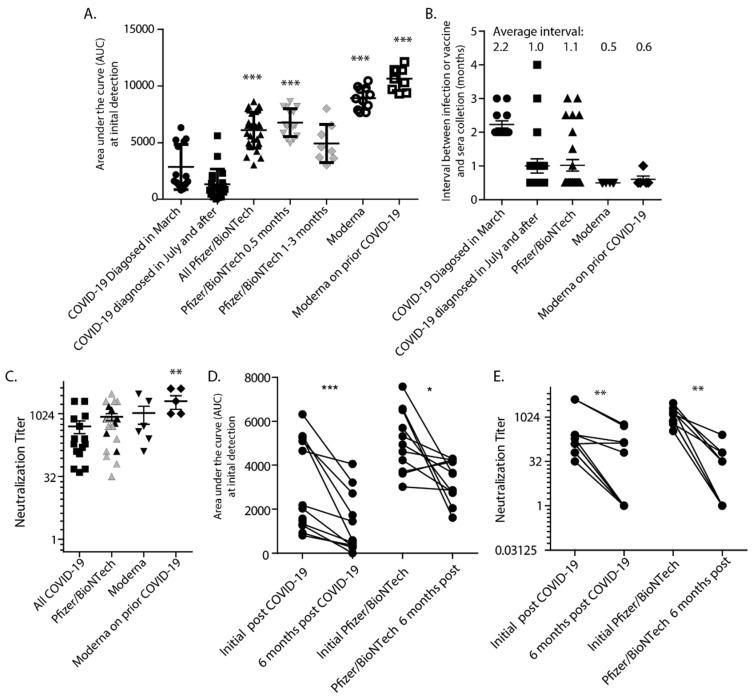
RBD-specific IgG and neutralization were compared between those that were naturally infected and those receiving COVID-19 vaccines. (**A**) Antibodies that were induced by vaccination were significantly higher levels than those initially induced by natural infections. Statistics are based on combined natural infections compared to vaccinations. *** *p* < 0.001 as determined by Kruskal-Wallis test with Dunns post-test. (**B**) The interval between serum collection and natural infection or vaccination was comparable. Notably, sera collected from those infected in March had a longer interval compared to those collected in July or later, but retained a similar average AUC. (**C**) The antibodies induced by the vaccines had slightly, but not significantly, higher neutralization capacity than those induced by natural infection, ** *p* < 0.01 as determined by the non-parametric Mann Whitney test. For the Pfizer/BioNTech data, the titers for the 0.5-month interval are shown in gray, and the 1–3 month interval are shown in black. This analysis excluded samples from natural infections for which neutralization was not detected. (**D**) AUCs detected initially or after 6 months following natural infection or vaccination with Pfizer/BioNTech were compared. (**E**) Initial and 6-month neutralization titers were also compared. Differences in paired samples were calculated using the Wilcoxon matched-pairs signed-rank test * *p* < 0.05, ** *p* < 0.01, *** *p* < 0.001.

Protection from infection has been assessed for participants in vaccine clinical trials [5,6] and the comparison of antibody titers and neutralization capacity induced by the vaccines compared to those generated in response to natural infection is of interest. Thus, we have assessed sera from those fully vaccinated (at least two weeks after the second dose) with either the Pfizer/BioNTech (23 people) or Moderna (11 people) vaccines and compared these antibody levels to those generated following natural infection. AUC levels were significantly higher in sera from those that were vaccinated compared to following natural infection (Figure 5A). For the Pfizer data, we also show the means of the group collected 2 weeks after vaccination, and those collected 1–3 months after vaccination. These data show that the mean AUC 2 weeks after vaccination with Pfizer/BioNTech is lower than the Moderna mean, also assessed 2 weeks post-vaccination. However, no matter which interval post-vaccination is compared, there were no statistically significant differences between Pfizer/BioNTech and Moderna, both vaccines induce robust responses. The interval between serum collection and COVID-19 diagnosis or second dose of the vaccine was slightly different (Figure 5B). The average time after COVID-19 diagnosis was 2.2 months for those infected in March, and 1.0 month for those infected in July and after. However, the average AUC from March was higher despite this longer interval between infection and sampling. Thus, sampling closer to diagnosis was likely not the reason for the differences in AUCs between the groups. Sera was collected an average of 1.0 months after vaccination with Pfizer/BioNTech and 2 weeks after Moderna. Thus, our natural infection and vaccine-induced antibody levels are comparable and the interval after infection or vaccination is not the reason for significant differences in antibody titers.

Neutralization titers for those immunized with either vaccine were slightly higher but not significantly different than those with detectible neutralization titers who were naturally infected (Figure 5C). Thus, differences in neutralization were not to the extent that would be expected based on AUC levels. In contrast to natural infection, there was no correlation between AUC and neutralization titers following vaccination with Pfizer (Pearson r = −0.1277, *p* = 0.5811), but there was a moderate correlation for Moderna (Pearson r = 0.6017, *p* = 0.0385). In Figure 5D,E, antibody AUC and neutralization titers were compared at 1–2 months post-infection or vaccination (with Pfizer/BioNTech) and at the 6-month interval. AUC values in both groups declined, but the vaccine group remained higher than the naturally infected group. This difference was not necessarily reflected in neutralization titers. The neutralization titers 6 months after vaccination had dropped similarly to those 6 months after natural infection. At this 6 month interval, there was again no correlation between AUC and neutralization titers for the Pfizer vaccine (Pearson r = 0.4580, *p* = 0.2538). Since the Moderna vaccine was more recently available, similar 6 month data for this vaccine are not yet available. Thus, while the vaccines clearly induce a very strong antibody response, the capacity of these antibodies to neutralize is lower than would be expected based on the correlation between AUC and neutralization generated with sera from natural infection. Finally, Figure 5A,C suggests that when individuals with prior infection are vaccinated, their antibody levels may be higher and more neutralizing than vaccination alone. Altogether, our data suggest that people who were diagnosed with COVID-19 or have detectible antibodies resulting from natural infection may have low-level antibodies that are not necessarily neutralizing, robust, or long-lasting. This population will still greatly benefit from vaccination that induces a higher magnitude antibody response than natural infection.

## 4. Discussion

A number of studies have already been published regarding the antibody response to COVID-19. Our study is novel in a number of aspects. We determined a temporal difference in antibody responses in our community and measured antibody responses over twelve months, allowing for the calculation of a strong correlation between starting levels of antibodies and the duration of the response. We also directly compared the antibody responses of those vaccinated with two different vaccines to those of naturally infected participants. We utilized a highly sensitive and specific ELISA assay to determine the antibody levels in a population that was not necessarily diagnosed with COVID-19. We determined that most, but not all, participants with a clinical COVID-19 diagnosis had detectible neutralizing antibody titers and detected a small number of participants that had no known COVID-19 interactions and no diagnosis but had low titer non-neutralizing antibodies. A similar study also determined that a high proportion of asymptomatic patients did not develop neutralizing antibodies, or lost them quickly [28]. Many investigations involve severely ill or hospitalized individuals and not a majority of asymptomatic people [10,11,12,13,14]. In these studies, failure to detect an antibody response is rare [10,16]. The results of many of these studies are in agreement with each other and with our own; however, there are some discrepancies. Long et al. reported a high proportion of both symptomatic and asymptomatic patients that did not develop detectible antibody responses after 8 weeks [29], which does not fully agree with our results. Another study that offered a snapshot of a patient population found that 100% of recovered patients had antibodies [30]. Thus, there is clearly a need for continuing research in this area to reach a consensus concerning antibody responses to COVID-19. Potentially regional, temporal or strain differences in SARS-CoV-2 circulation play a role in these disparate findings.

Many other reports have also shown strong correlations between disease severity and antibody responses [10,11,12,13,14,15]. Thus, a strong inflammatory response to viral infection inducing severe disease generally results in a robust immune response to COVID-19 while a strong antibody response is not expected following asymptomatic SARS-CoV-2 infection. Dan et al. found that antibody responses were strongly predictive of memory B cell development, but less so for memory T cells, with a large amount of variation. The majority of people with SARS-CoV-2 specific antibody responses developed some component of immune memory. [16]. Wang et al. carefully measured memory T cell responses in individuals diagnosed with COVID-19 and their close contacts. Memory T cells were detected even in contacts that tested negative for SARS-CoV-2, indicating that this branch of immunity is capable of developing even in the absence of robust antibody responses [31]. However, others showed that T cell responses correlated with SARS-CoV-2 neutralization [26]. The extent to which T cells contribute to protection from SARS-CoV-2 infection remains to be determined.

Following the same participants for 12 months has allowed us to determine a strong correlation between the starting antibody levels and the duration of the antibody response. Furthermore, our data suggest that the vaccines induce a much stronger antibody response than does natural infection, although the correlation between AUC and neutralization may not be the same. Our data also suggest that the Moderna vaccine boosts existing responses from natural infection, which is likely the case for all vaccines. Because memory B cells and other immune memory components also develop when circulating antibodies can be detected [16], SARS-CoV-2 vaccine-induced immunity is likely to be very long-lasting as it is for other viral infections and vaccines. Indeed, memory B cells with the capacity to neutralize the strain responsible for the 1918 influenza outbreak have been detected in survivors in their 90s and 100s [32]. Altogether, because of the variability of antibody responses following natural infection, these data underscore the importance of vaccination even for people with a prior positive diagnosis of SARS-CoV-2 infection or antibody detection to achieve herd immunity.

## Figures and Tables

**Table 1:** Numbers of unvaccinated participants and disease severity.

**Participant Group**	**Enrolled**	**ELISA Positive**	**Neutralization Positive**	**Asymptomatic**	**Mild**	**Medium**	**Severe**	**Required** **Hospitalization**
Diagnosed with SARS-CoV-2 infection or known close contact March 2020	14	14	14	1	6	4	3	1
Diagnosed with SARS-CoV-2 infection July 2020 or later	20	15	16 ^a^	0	12	5	3	0
No known diagnosis or contact	134	10	2	6 ^b^	2 ^b^	0 ^b^	2 ^b^	0
Total participants ^c^	168	39	32	7	18	9	6	1

^a^ One sera was under the ELISA cutoff but retained neutralization capacity. ^b^ Of the 10 people who had detectible anti-RBD IgG. ^c^ 107 females, 61 males.

## Data Availability

The data presented in this study are openly available at FigShare at https://doi.org/10.6084/m9.figshare.14710917.v1, accessed on 31 May 2021.

## References

[1] Addetia A., Crawford K.H.D., Dingens A., Zhu H., Roychoudhury P., Huang M.-L., Jerome K.R., Bloom J.D., Greninger A.L. (2020). Neutralizing antibodies correlate with protection from SARS-CoV-2 in humans during a fishery vessel outbreak with a high attack rate. J. Clin. Microbiol..

[2] Deng W., Bao L., Liu J., Xiao C., Liu J., Xue J., Lv Q., Qi F., Gao H., Yu P. (2020). Primary exposure to SARS-CoV-2 protects against reinfection in rhesus macaques. Science.

[3] Chandrashekar A., Liu J., Martinot A.J., McMahan K., Mercado N.B., Peter L., Tostanoski L.H., Yu J., Maliga Z., Nekorchuk M. (2020). SARS-CoV-2 infection protects against rechallenge in rhesus macaques. Science.

[4] Hassan A.O., Case J.B., Winkler E.S., Thackray L.B., Kafai N.M., Bailey A.L., McCune B.T., Fox J.M., Chen R.E., Alsoussi W.B. (2020). A SARS-CoV-2 Infection Model in Mice Demonstrates Protection by Neutralizing Antibodies. Cell.

[5] Polack F.P., Thomas S.J., Kitchin N., Absalon J., Gurtman A., Lockhart S., Perez J.L., Pérez Marc G., Moreira E.D., Zerbini C. (2020). Safety and efficacy of the BNT162b2 mRNA COVID-19 vaccine. N. Engl. J. Med..

[6] Sahin U., Muik A., Derhovanessian E., Vogler I., Kranz L.M., Vormehr M., Baum A., Pascal K., Quandt J., Maurus D. (2020). COVID-19 vaccine BNT162b1 elicits human antibody and TH1 T cell responses. Nature.

[7] Mulligan M.J., Lyke K.E., Kitchin N., Absalon J., Gurtman A., Lockhart S., Neuzil K., Raabe V., Bailey R., Swanson K.A. (2020). Phase I/II study of COVID-19 RNA vaccine BNT162b1 in adults. Nature.

[8] Baden L.R., El Sahly H.M., Essink B., Kotloff K., Frey S., Novak R., Diemert D., Spector S.A., Rouphael N., Creech C.B. (2020). Efficacy and Safety of the mRNA-1273 SARS-CoV-2 Vaccine. N. Engl. J. Med..

[9] Wolf J., Abzug M.J., Wattier R.L., Sue P.K., Vora S.B., Zachariah P., Dulek D.E., Waghmare A., Olivero R., Downes K.J. (2021). Initial guidance on use of monoclonal antibody therapy for treatment of COVID-19 in children and adolescents. J. Pediatric. Infect. Dis. Soc..

[10] Zhao J., Yuan Q., Wang H., Liu W., Liao X., Su Y., Wang X., Yuan J., Li T., Li J. (2020). Antibody responses to SARS-CoV-2 in patients of novel coronavirus disease 2019. Clin. Infect. Dis..

[11] Hansen C.B., Jarlhelt I., Pérez-Alós L., Hummelshøj Landsy L., Loftager M., Rosbjerg A., Helgstrand C., Bjelke J.R., Egebjerg T., Jardine J.G. (2020). SARS-CoV-2 antibody responses determine disease severity in COVID-19 infected individuals. medRxiv.

[12] Röltgen K., Powell A.E., Wirz O.F., Stevens B.A., Hogan C.A., Najeeb J., Hunter M., Wang H., Sahoo M.K., Huang C. (2020). Defining the features and duration of antibody responses to SARS-CoV-2 infection associated with disease severity and outcome. Sci. Immunol..

[13] Dispinseri S., Secchi M., Pirillo M.F., Tolazzi M., Borghi M., Brigatti C., De Angelis M.L., Baratella M., Bazzigaluppi E., Venturi G. (2021). Neutralizing antibody responses to SARS-CoV-2 in symptomatic COVID-19 is persistent and critical for survival. Nat. Commun..

[14] Lau E.H.Y., Tsang O.T.Y., Hui D.S.C., Kwan M.Y.W., Chan W.-H., Chiu S.S., Ko R.L.W., Chan K.H., Cheng S.M.S., Perera R.A.P.M. (2021). Neutralizing antibody titres in SARS-CoV-2 infections. Nat. Commun..

[15] Brochot E., Demey B., Touzé A., Belouzard S., Dubuisson J., Schmit J.-L., Duverlie G., Francois C., Castelain S., Helle F. (2020). Anti-spike, anti-nucleocapsid and neutralizing antibodies in SARS-CoV-2 inpatients and asymptomatic individuals. Front. Microbiol..

[16] Dan J.M., Mateus J., Kato Y., Hastie K.M., Yu E.D., Faliti C.E., Grifoni A., Ramirez S.I., Haupt S., Frazier A. (2021). Immunological memory to SARS-CoV-2 assessed for up to 8 months after infection. Science.

[17] Gaebler C., Wang Z., Lorenzi J.C.C., Muecksch F., Finkin S., Tokuyama M., Cho A., Jankovic M., Schaefer-Babajew D., Oliveira T.Y. (2021). Evolution of antibody immunity to SARS-CoV-2. Nature.

[18] Fill Malfertheiner S., Brandstetter S., Roth S., Harner S., Buntrock-Döpke H., Toncheva A.A., Borchers N., Gruber R., Ambrosch A., Kabesch M. (2020). Immune response to SARS-CoV-2 in health care workers following a COVID-19 outbreak: A prospective longitudinal study. J. Clin. Virol..

[19] Seow J., Graham C., Merrick B., Acors S., Pickering S., Steel K.J.A., Hemmings O., O’Byrne A., Kouphou N., Galao R.P. (2020). Longitudinal observation and decline of neutralizing antibody responses in the three months following SARS-CoV-2 infection in humans. Nat. Microbiol..

[20] Anand S., Montez-Rath M., Han J., Bozeman J., Kerschmann R., Beyer P., Parsonnet J., Chertow G.M. (2020). Prevalence of SARS-CoV-2 antibodies in a large nationwide sample of patients on dialysis in the USA: A cross-sectional study. Lancet.

[21] Higgins R.L., Rawlings S.A., Case J., Lee F.Y., Chan C.W., Barrick B., Burger Z.C., Yeo K.-T.J., Marrinucci D. (2021). Longitudinal SARS-CoV-2 antibody study using the Easy Check COVID-19 IgM/IgG™ lateral flow assay. PLoS ONE.

[22] Stadlbauer D., Amanat F., Chromikova V., Jiang K., Strohmeier S., Arunkumar G.A., Tan J., Bhavsar D., Capuano C., Kirkpatrick E. (2020). SARS-CoV-2 seroconversion in humans: A detailed protocol for a serological assay, antigen production, and test setup. Curr. Protoc. Microbiol..

[23] Hedges J.F., Balasuriya U.B., Timoney P.J., McCollum W.H., MacLachlan N.J. (1999). Genetic divergence with emergence of novel phenotypic variants of equine arteritis virus during persistent infection of stallions. J. Virol..

[24] Loveday E.K., Hain K.S., Kochetkova I., Hedges J.F., Robison A., Snyder D.T., Brumfield S.K., Young M.J., Jutila M.A., Chang C.B. (2020). Effects of inactivation method on SARS-CoV-2 virion protein and structure. bioRxiv.

[25] Amanat F., Stadlbauer D., Strohmeier S., Nguyen T.H.O., Chromikova V., McMahon M., Jiang K., Arunkumar G.A., Jurczyszak D., Polanco J. (2020). A serological assay to detect SARS-CoV-2 seroconversion in humans. Nat. Med..

[26] Ni L., Ye F., Cheng M.-L., Feng Y., Deng Y.-Q., Zhao H., Wei P., Ge J., Gou M., Li X. (2020). Detection of SARS-CoV-2-specific humoral and cellular immunity in COVID-19 convalescent individuals. Immunity.

[27] Winkler E.S., Bailey A.L., Kafai N.M., Nair S., McCune B.T., Yu J., Fox J.M., Chen R.E., Earnest J.T., Keeler S.P. (2020). SARS-CoV-2 infection of human ACE2-transgenic mice causes severe lung inflammation and impaired function. Nat. Immunol..

[28] Lei Q., Li Y., Hou H.Y., Wang F., Ouyang Z.Q., Zhang Y., Lai D.Y., Banga Ndzouboukou J.L., Xu Z.W., Zhang B. (2020). Antibody dynamics to SARS-CoV-2 in asymptomatic COVID-19 infections. Allergy.

[29] Long Q.-X., Tang X.-J., Shi Q.-L., Li Q., Deng H.-J., Yuan J., Hu J.-L., Xu W., Zhang Y., Lv F.-J. (2020). Clinical and immunological assessment of asymptomatic SARS-CoV-2 infections. Nat. Med..

[30] Wang X., Guo X., Xin Q., Pan Y., Hu Y., Li J., Chu Y., Feng Y., Wang Q. (2020). Neutralizing antibody responses to severe acute respiratory syndrome coronavirus 2 in coronavirus disease 2019 inpatients and convalescent patients. Clin. Infect. Dis..

[31] Wang Z., Yang X., Zhong J., Zhou Y., Tang Z., Zhou H., He J., Mei X., Tang Y., Lin B. (2021). Exposure to SARS-CoV-2 generates T-cell memory in the absence of a detectable viral infection. Nat. Commun..

[32] Yu X., Tsibane T., McGraw P.A., House F.S., Keefer C.J., Hicar M.D., Tumpey T.M., Pappas C., Perrone L.A., Martinez O. (2008). Neutralizing antibodies derived from the B cells of 1918 influenza pandemic survivors. Nature.

